# Functional and transcriptomic insights into 46,XY disorders of sex development associated with *NR5A1* gene variants

**DOI:** 10.1186/s13293-026-00939-0

**Published:** 2026-06-09

**Authors:** Qingxu Liu, Shaolian Zang, Yan Li, Xiaoqin Yin, Pin Li

**Affiliations:** Department of Endocrinology, Shanghai Children’s Hospital, School of Medicine, Shanghai Jiao Tong University, Shanghai, 200062 People’s Republic of China

**Keywords:** *NR5A1*, 46, XY DSD, disorders of sexual development, *STARD8*, *AMHR2*, steroid hormone, transcription

## Abstract

**Background:**

*NR5A1* encodes a transcription factor essential for adrenal and gonadal development. Gene variants are a known cause of heterogeneous 46,XY disorders of sex development (DSD), but the mechanisms underlying the phenotypic variability remain unclear. We investigated how different *NR5A1* variants affect downstream gene regulation and contribute to DSD pathogenesis.

**Methods:**

We analyzed four naturally occurring *NR5A1* variants identified in patients with 46,XY DSD—two novel (p.Cys65Ser, p.His310Arg) and two previously reported (p.Cys30Ser, p.Gln329*). We performed protein and transcriptomic analyses to characterize variant effects and identify dysregulated and candidate target genes, validated by qPCR and luciferase assays. Transcriptomic and CUT&Tag analyses focused on the p.Gln329* truncating variant.

**Results:**

All four variants occurred at conserved residues and resulted in reduced NR5A1 protein expression and impaired nuclear localization upon transfection in HEK293T cells. Transcriptomic analysis using the p.Gln329* variant revealed broad downregulation of genes involved in steroidogenesis, including *CYP11A1*, *STAR*, and *CYP17A1*. Notably, *AMHR2* and *STARD8* were significantly downregulated and showed reduced CUT&Tag signal in variant-transfected cells. Promoter assays confirmed that all variants diminished *CYP11A1* and *AMHR2* promoter activity. Only the p.Gln329* variant affected *STARD8* promoter activity.

**Conclusions:**

These findings indicate that *NR5A1* variants impair protein expression and localization, leading to transcriptional dysregulation of genes involved in steroid hormone biosynthesis and sexual development. Based on analysis of the p.Gln329* truncating variant, *AMHR2* and *STARD8* are strong candidate novel downstream targets of NR5A1, offering further insight into the mechanisms driving 46,XY DSD.

**Supplementary Information:**

The online version contains supplementary material available at 10.1186/s13293-026-00939-0.

## Abstract (plain language summary)

Differences in sex development can occur when the genetic instructions for building male reproductive organs are disrupted before birth. This study focuses on a key gene called *NR5A1*, which acts like a master switch to turn on other genes essential for testis development and hormone production. We investigated four variants in this gene found in children with 46,XY differences in sex development. Using cell models, we showed that these variants cause the NR5A1 protein to be produced at lower levels and prevent it from reaching the cell’s nucleus, where it normally works. As a result, the variant proteins fail to properly switch on several crucial target genes involved in making male hormones. We identified two new candidate genes controlled by NR5A1, called *AMHR2* and *STARD8*, which may play important roles in this process. Our research helps explain how changes in a single gene can lead to a broad range of developmental outcomes and provides a clearer picture of the molecular steps involved in human sex development.

**Supplementary Information:**

The online version contains supplementary material available at 10.1186/s13293-026-00939-0.

## Highlights

- Four *NR5A1* variants, including two novel ones (p.Cys65Ser, p.His310Arg), were identified in patients with 46,XY DSD and severe genital anomalies.
- All variants reduced NR5A1 protein expression and disrupted its normal localization to the nucleus.
- Transcriptomic analysis using the p.Gln329* variant revealed broad downregulation of steroidogenic genes, including *CYP11A1*, *STAR*, and *CYP17A1*.
- *AMHR2* and *STARD8* were identified as candidate transcriptional targets of NR5A1.
- Differential dysregulation of these candidate genes, particularly *STARD8*, provides potential mechanistic clues for the variable phenotypic severity in 46,XY DSD.

**Supplementary Information:**

The online version contains supplementary material available at 10.1186/s13293-026-00939-0.

## Background

The *NR5A1* (Nuclear Receptor Subfamily 5 Group A Member 1) gene is located on chromosome 9q33 and encodes a protein of 461 amino acids. NR5A1 encodes a transcription factor with four key functional domains: a DNA-binding domain (DBD), an auxiliary hinge region, a ligand-binding domain (LBD), and two activation function domains (AF-1 and AF-2). In male mice, *Nr5a1* is expressed in both Leydig and Sertoli cells [1], with its expression preceding that of *Sry*, the gene that initiates testicular development. By embryonic day 12.5 (E12.5), testicular cords begin to form, and *Nr5a1* transcripts can be detected throughout gonadal differentiation [2]. As a core regulator of steroidogenic gene expression, NR5A1 is essential for steroid hormone biosynthesis.Through its functional domains, NR5A1 participates in key biological processes, including gonadal and adrenal development, steroid hormone biosynthesis, and reproduction. In Sertoli cells, NR5A1 activates *AMH* expression starting around the seventh gestational week, inducing regression of Müllerian ducts during male fetal development. It is also essential for Sertoli cells proliferation and survival [3, 4]; *NR5A1* variants reduce these processes, leading to decreased Sertoli cell numbers. In fetal Sertoli cells, NR5A1 cooperates with *SRY* and *SOX9* to drive *SOX9* transcription and maintain *AMH* expression. In Leydig cells, NR5A1 activates steroidogenic enzymes necessary for masculinization of external genitalia. It induces *CYP11A1* expression [5] and increases insulin-like peptide-3, both essential for testosterone biosynthesis and testicular descent. Variants in *NR5A1* are inherited in an autosomal dominant manner and account for approximately 10–15% of 46,XY disorders of sex development (DSD) [6]. The clinical phenotypes associated with *NR5A1* variants are diverse and heterogeneous, with reported racial differences. Although 46,XY gonadal dysgenesis is the most common clinical manifestation, most identified variants cannot be clearly linked to specific phenotypes [7, 8]. *NR5A1*-related DSD in humans is rarely associated with adrenal insufficiency [9], suggesting that many variants disrupt gonadal development pathways without impairing adrenal function. The extent to which different *NR5A1* variants alter steroidogenic and sex-determining gene networks remains to be fully elucidated.

## Methods

### Ethical approval

All procedures were ethically approved by the Ethics Committee of Shanghai Children’s Hospital, Shanghai Jiao Tong University (approval number: 2021R092-E01).

### Patients

Four patients in the Department of Endocrinology of Shanghai Children’s Hospital from 2018 to 2023 were studied. We investigated the clinical manifestations, family history, related sex hormones and *NR5A1* gene sequences of the 46,XY patients. We evaluated the external genital phenotypes according to the external masculinization score. Phallus lengths were compared with data from normal Chinese children. Cryptorchidism was confirmed by physical examination and ultrasound examination, in line with the diagnostic criteria for pediatric cryptorchidism. Cryptorchidism was classified as intra-abdominal cryptorchidism or inguinal cryptorchidism according to the location of the testis. The presence or absence of Müllerian structures was assessed by ultrasound. *NR5A1* genetic test results were collected from four patients carrying *NR5A1* variants. The c.193T > A (p.Cys65Ser) and c.929 A > G (p.His310Arg) variants were novel, and the c.88T > A (p.Cys30Ser) and c.985 C > T (p.Gln329*) had been reported previously [10] but lacked functional validation.

### Hormonal analysis

To evaluate hypothalamic-pituitary-gonadal axis function and testicular function, all patients underwent gonadotropin-releasing hormone stimulation and human chorionic gonadotropin (HCG) stimulation. Sex hormones included anti-Müllerian hormone (AMH), inhibin B, basal testosterone (T), basal dihydrotestosterone (DHT), basal luteinizing hormone (LH), peak LH, basal follicle-stimulating hormone (FSH), peak FSH, DHT (and T) after HCG stimulation. Serum AMH and inhibin B were tested using solid-phase sandwich enzyme-linked immunosorbent assays (ELISAs) purchased from Guangzhou Kangrun Biotechnology Co., Ltd. T and DHT were detected by ELISA and measured using a Polar ELx800 microplate reader (USA). Serum LH and FSH concentrations were tested with LH and FSH detection kits (Beckman Coulter) and measured with an automatic immunoluminescence analyser (UnicelDxI 800).

### Classification of *NR5A1* variants

Variant interpretation included database searches using ClinVar (ClinVar, https://www.ncbi.nlm.nih.gov/clinvar/) and the Human Gene Mutation Database (http://www.hgmd.cf.ac.uk). In silico pathogenicity predictions were obtained using PROVEAN, SIFT, and Polyphen-2. Variant classification was conducted according to American College of Medical Genetics and Genomics (ACMG) guidelines. Conservation of amino acid residues across species was assessed using the UniProt (https://www.uniprot.org/) database. Protein structure modeling was performed using SWISS-MODEL (https://www.swissmodel.expasy.org/), and predicted structural changes in the protein structure of NR5A1 variations were visualized using PyMOL (https://pymol.org/2/).

### Construction and sequencing verification of mutated plasmids

The full-length synthesized commercially *NR5A1* gene was subjected to enzymatic digestion, and the target fragment was recovered and ligated into a lentiviral expression vector. Cloning was carried out in competent *E. coli* DH5α cells. Colonies were selected on LB plates containing the appropriate antibiotic. Plasmid DNA was extracted, digested to confirm insert presence, and verified by Sanger sequencing. Each variant was later introduced by site direct mutagenesis.

### Cell culture

HEK293T cells were purchased from the Cell Bank of the Chinese Academy of Sciences (Shanghai, China). The cells were maintained in Dulbecco’s modified Eagle’s medium (DMEM, Gibco, NY, USA) supplemented with 10% foetal bovine serum (FBS, Gibco). The cells were cultured in a humidified atmosphere containing 5% CO2 at 37 °C.

### Western blotting

HEK293T cells were transfected with wild-type, mutant, or empty vector plasmids. After 48 h, whole-cell lysates were collected using RIPA lysis buffer (Cat# P0013B, Beyotime, China). After SDS‒PAGE, immunoblotting was performed with polyclonal NR5A1 antibody (1:600, Cat# 18658-1-AP, Proteintech, USA) and an anti-GAPDH antibody. Each experiment was repeated three times. Band intensities were quantified using ImageJ software to assess differences in protein expression levels.

### Immunocytochemistry

HEK293T cells were transfected with wild-type (WT) or mutant *NR5A1* plasmids, or empty vector. After 48 h, the plasmids were transfected into HEK293T cells seeded in a confocal dish with Liposomal Transfection Reagent. After 24 h, the medium was removed, and the cells were fixed with 4% paraformaldehyde fixative solution for 15 min. Nuclear counterstaining was performed with 4,6-diamidino-2-phenylindole (DAPI)(Cat# P0131, Beyotime, China) for 5 min. Images were obtained via confocal laser scanning microscopy (Leica, Germany). Quantitative analysis of subcellular localization was not performed; evaluation was based on qualitative visual assessment of multiple fields from three independent experiments.

### Transcriptome sequencing, data processing, and analysis

Total RNA was extracted from transfected HEK293T cells using the TRIzol reagent (Invitrogen, CA, USA) according to the manufacturer’s protocol. Three independent biological replicates were prepared for each condition. RNA purity and quantification were evaluated using the NanoDrop 2000 spectrophotometer (Thermo Scientific, USA). RNA integrity was assessed using the Agilent 2100 Bioanalyzer (Agilent Technologies, Santa Clara, CA, USA). Then the libraries were constructed using VAHTS Universal V6 RNA-seq Library Prep Kit according to the manufacturer’s instructions. The transcriptome sequencing and analysis were conducted by OE Biotech Co., Ltd. (Shanghai, China). Sequencing was performed on an Illumina NovaSeq 6000 platform, generating 150 bp paired-end reads. Raw reads of fastq format were firstly processed using fastp and the low-quality reads were removed to obtain the clean reads. Genes with |log₂(fold change)| ≥ 1 and adjusted *P* ≤ 0.05 were considered differentially expressed. For identification of significantly dysregulated genes, a more stringent threshold of |log₂(fold change)| ≥ 1.5 and adjusted *P* ≤ 0.05 was applied. The less stringent cutoff was used to capture broad pathway changes for GO/KEGG enrichment, while the more stringent cutoff was applied to select robust candidate targets for focused validation. Functional annotation of differentially expressed genes was performed using Gene Ontology (GO) and Kyoto Encyclopedia of Genes and Genomes (KEGG) pathway enrichment, analyzed with the *clusterProfiler* package. GO terms were categorized by biological process, cellular component, and molecular function [11].

### CUT&Tag sequencing, data processing, and analysis

The CUT&Tag (Cleavage Under Targets and Tagmentation) assay was prepared using the Hyperactive™ In-Situ ChIP Library Prep Kit for Illumina (Cat# TD901-TD902, Vazyme Biotech, China) according to manufacturer’s instruction. Three independent biological replicates were used. Briefly, prepared concanavalin A-coated magnetic beads (ConA beads) were added to resuspended cells and incubated at room temperature to bind cells. Non-ionic detergent Digitonin was used to permeate cell membrane. Then, primary anti-NR5A1 antibody (Cat# 18658-1-AP, Proteintech Group, Inc.), secondary antibody and the Hyperactive pA-Tn5 Transposase were incubated with the cells that were bounded by ConA beads in order. Therefore, the Hyperactive pA-Tn5 Transposase can exactly cut off the DNA fragments that were bound with target protein. In addition, the cut DNA fragments can be ligated with P5 and P7 adaptors by Tn5 transposase and the libraries were amplified by PCR with the P5 and P7 primers. The purified PCR products were evaluated using the Agilent 2100. Bioanalyzer (Agilent Technologies, Santa Clara, CA, USA). Finally, these libraries were sequenced on the Illumina NovaSeq6000 platform and 150 bp paired-end reads were generated for the following analysis. The raw sequence data were firstly quality trimmed by fastp software to obtain the clean reads. Then the clean reads were aligned to the human genome GRCh38.p12 using Bowtie2 and subsequently analyzed by the SEACR software based on the ‘stringent’ parameter to detect genomic regions enriched for multiple overlapping DNA fragments (peaks) that we considered to be putative binding sites. The statistically significant differential binding sites were identified based on binding affinity with the threshold of *P* < 0.05 and |log2(Fold)|>1. A list of significantly differentially expressed genes was first generated from RNA sequencing analysis. CUT&Tag binding peaks were annotated and analyzed using bedtools to identify regions overlapping with gene transcription start sites, defined as ± 3 kb from the TSS. The resulting gene list was recorded and annotated. Visualization of peak distribution along genomic regions of interested genes was performed with Integrative Genomics Viewer (IGV).

### Fluorescence quantitative PCR

Total RNA was extracted from (HEK293T cells transfected with the *NR5A1* WT or mutant plasmids) using mirVana™ RNA Isolation Kit (Cat# AM1561) according to the manufacturer’s specifications. The yield of RNA was determined using a NanoDrop 2000 spectrophotometer (Thermo Scientific, USA), and the integrity was evaluated using agarose gel electrophoresis stained with ethidium bromide. Quantification was performed with a two-step reaction process: reverse transcription (RT) and PCR. Each RT reaction consisted of 0.5 µg RNA, 2 µl of 5×TransScript All-in-one SuperMix for qPCR and 0.5 µl of gDNA Remover, in a total volume of 10 µl. Reactions were performed in a GeneAmp^®^ PCR System 9700 (Applied Biosystems, USA) for 15 min at 42℃, 5 s at 85℃.The 10 µl RT reaction mix was then diluted 10-fold in nuclease-free water and held at -20℃. Real-time PCR was performed using LightCycler^®^ 480 Ⅱ Real-time PCR Instrument (Roche, Swiss) with 10 µl PCR reaction mixture that included 1 µl of cDNA, 5 µl of 2×PerfectStartTM Green qPCR SuperMix, 0.2 µl of forward primer, 0.2 µl of reverse primer and 3.6 µl of nuclease-free water. Reactions were incubated in a 384-well optical plate (Roche, Swiss) at 94℃ for 30 s, followed by 45 cycles of 94℃ for 5 s, 60℃ for 30 s. Each sample was run in triplicate for analysis. At the end of the PCR cycles, melting curve analysis was performed to validate the specific generation of the expected PCR product. The primer sequences were designed in the laboratory and synthesized by TsingKe Biotech based on the mRNA sequences obtained from the NCBI database as follows: For *STARD8*, the upstream primer is GTCACCACGAGCTGTTTC, and the downstream primer is TCCACTCTCACATCCCTATCT. For *AMHR2*, the upstream primer is CTTGACCCAGTACACCAGTGA, and the downstream primer is ACCTGGTTTATATTGGCCATTC. The expression levels of mRNAs were normalized to *GAPDH* and *ACTB* and were calculated using the 2^−ΔΔCt^ method.

### Dual-luciferase reporter assay

HEK293T cells were co-transfected with the *NR5A1* WT or mutant plasmids, along with the pGL3-basic firefly luciferase reporter, pGL3-control, and pRL-TK Renilla luciferase plasmid as an internal control. Vector construction was performed on three differentially expressed gene promoters and three controls that appeared in mRNA sequencing. The *AMHR2* promoter, *CYP11A1* promoter, and *STARD8* promoter were constructed onto a pGL3 basic vector. The primers for the three gene promoters, *AMHR2*, *CYP11A1*, and *STARD8*, used are listed in Table S1. HEK 293 T cells were plated in 96-well plates and cotransfected with the *NR5A1* wild-type or mutant overexpression plasmid (200 ng/well), firefly luciferase reporter (*AMHR2* promoter, *CYP11A1* promoter, or *STARD8* promoter )(200 ng/well), or Renilla luciferase reporter pRL-TK (200 ng/well), which served as the internal reference, using Lipo8000™ Transfection Reagent (Cat# C0533, Beyotime Biotechnology, China). Luciferase activity was measured with a Dual-Lumi™ Dual-Luciferase Reporter Gene Assay Kit (Cat# RG088S, Beyotime Biotechnology, China) according to the manufacturer’s instructions. Firefly luciferase activity was normalized to Renilla luciferase activity and presented as relative luciferase activity. The samples were measured in triplicate in each assay, and all the assays were independently repeated more than three times.

### Statistical analyses

All statistical analyses were performed using SPSS 26.0, and graphs were generated using GraphPad Prism 9.0. Data from functional experiments are presented as means ± standard deviation (SD). For comparisons involving three or more groups, one-way analysis of variance (ANOVA) was used. Statistical significance was defined as *P* < 0.05, with significance levels indicated as follows: *P* < 0.05; ** indicating *P* < 0.01; *** indicating *P* < 0.001; **** indicating *P* < 0.0001.

## Results

### Patient phenotypes and *NR5A1* variants characterization

To explore the phenotypic consequences of NR5A1 mutations in 46,XY DSD, we analyzed four patients harboring distinct heterozygous variants: p.Cys30Ser, p.Cys65Ser, p.His310Arg, and p.Gln329*. All four variants were identified through whole-exome sequencing and selected for further functional and transcriptomic investigation based on their predicted impact on key NR5A1 protein domains. We first assessed the clinical features associated with each variant and performed in silico pathogenicity predictions to evaluate their likely deleteriousness. All four patients with 46,XY DSD due to *NR5A1* variants presented with severe hypospadias (abnormal placement of the urethral opening) and bifid scrotum (incomplete scrotal fusion). Three also had inguinal cryptorchidism (undescended testes), while none exhibited isolated micropenis or cryptorchidism. The primary clinical presentation was partial gonadal dysgenesis, and low External Masculinization Scores (EMS) reflected the severity of the phenotype. In silico predictions for all point mutations indicated pathogenicity (Table 1). Comprehensive endocrine profiles and follow-up gender are provided in Table 2.

**Table 1:** **Clinical characteristics**, ***NR5A1*** **gene variants**,** and in silico pathogenicity predictions in four patients with 46**,**XY DSD**

Case	Age (years)	Gender	Proximal Hypospadias	Bifid scrotum	Cryptorchidism	EMS	Variant	Amino acid changes	Domain	Type	Novel	Pathogenicity	Provean	SIFT	Polyphen-2	Inheritance
N1	1	M	Y	Y	Inguinal	2.5	c.88T > A	p.Cys30Ser	DBD	Missense	N	LP	-8.22	0.0	0.988	Spontaneous
N2	2.5	F	Y	Y	Inguinal	2	c.193T > A	p.Cys65Ser	DBD	Missense	Y	LP	-8.73	0.045	1.0	Spontaneous
N3	2.4	F	Y	Y	Scrotum	3	c.929 A > G	p.His310Arg	LBD	Missense	Y	VUS	-5.59	0.005	0.998	Mother
N4	1.5	M	Y	Y	Inguinal	2	c.985 C > T	p.Gln329*	LBD	Nonsense	N	LP	U	U	U	Spontaneous

Variant classification was based on ACMG guidelines, and pathogenicity was assessed using Provean, SIFT, and PolyPhen-2. Inheritance was determined by parental testing. Abbreviations: EMS, External Masculinization Score; DBD, DNA-binding domain; LBD, Ligand-binding domain; LP, Likely pathogenic; VUS, Variant of uncertain significance; U, Unknown

### Hormones

To evaluate the integrity of the hypothalamic‑pituitary‑gonadal axis and testicular function, all four patients underwent HCG stimulation testing. All four patients exhibited elevated basal FSH levels (3.02–8.21 IU/L), substantially exceeding the normal prepubertal male reference range (e.g., ≤ 1.9 IU/L for ages 1–5 years per Mayo Clinic Laboratories), supporting the diagnosis of primary gonadal insufficiency. Consistent with this, FSH rose further after stimulation (peak 17.1–37.15 IU/L). Basal LH levels ranged from 0.37 to 1.02 IU/L, within or near the upper limit of the normal prepubertal reference range (e.g., ≤ 1.3 IU/L for ages 1–6 years per Mayo Clinic Laboratories).

Sertoli cell function, reflected by AMH and inhibin B, was differentially affected. AMH was measurable in all patients, ruling out complete Sertoli cell agenesis, but levels varied widely (11.82 to 46.45 pmol/L). Patient N2 (p.Cys65Ser) had the lowest AMH (11.82 pmol/L) and low inhibin B (80.47 pg/mL), suggesting the most severe Sertoli cell impairment, consistent with his low EMS (2.0). Patient N3 (p.His310Arg), who had the highest EMS (3.0), showed the best‑preserved inhibin B (225.53 pg/mL).

After HCG stimulation, testosterone increased in all patients, but the magnitude of response varied considerably. DHT levels increased appropriately post‑HCG, with particularly robust responses in N1 and N2 (78.59 and 147.92 pg/mL, respectively), indicating intact 5α‑reductase activity and that undervirilisation results primarily from insufficient androgen synthesis rather than impaired peripheral conversion.

Two patients (N2 and N3) were initially assigned female sex due to severe genital ambiguity but were subsequently reassigned and raised as male following endocrine confirmation of functional testicular tissue and adequate androgen response to HCG. No Müllerian structures were identified by ultrasound in any patient. This finding is consistent with the presence of measurable AMH in all cases; even the lowest AMH level observed in patient N2 (11.82 pmol/L) appears to have been sufficient during fetal life to induce complete regression of the Müllerian ducts. This observation aligns with the current understanding that relatively low concentrations of AMH are required for Müllerian regression, and that AMH bioactivity may remain intact despite quantitative reductions. The absence of Müllerian remnants further supported the decision to raise all patients as male (Table 2).

**Table 2:** **Hormone levels**,** Müllerian structures and follow‑up gender of the four patients**

Case	AMH (pmol/L)	Inhibin B (pg/mL)	Basal LH (IU/L)	Peak LH (IU/L)	Basal FSH (IU/L)	Peak FSH (IU/L)	Basal T (nmol/L)	T after HCG stimulation (nmol/L)	Basal DHT (pg/mL)	DHT after HCG stimulation (pg/mL)	Müllerian structure	Follow‑up gender
N1	46.45	160.24	0.37	10.7	3.02	17.1	0.69	2.87	59.94	78.59	absence	M
N2	11.82	80.47	0.68	12.27	8.21	37.15	0.38	3.54	103.19	147.92	absence	M
N3	22.86	225.53	0.8	14.93	5.97	19.02	0.35	3.74	33.23	31.47	absence	M
N4	18	113.46	1.02	18.18	5.81	23.83	1.96	8.79	14.83	62.96	absence	M

### Analysis of conservation and structural changes of *NR5A1* variants

**Fig. 1:**
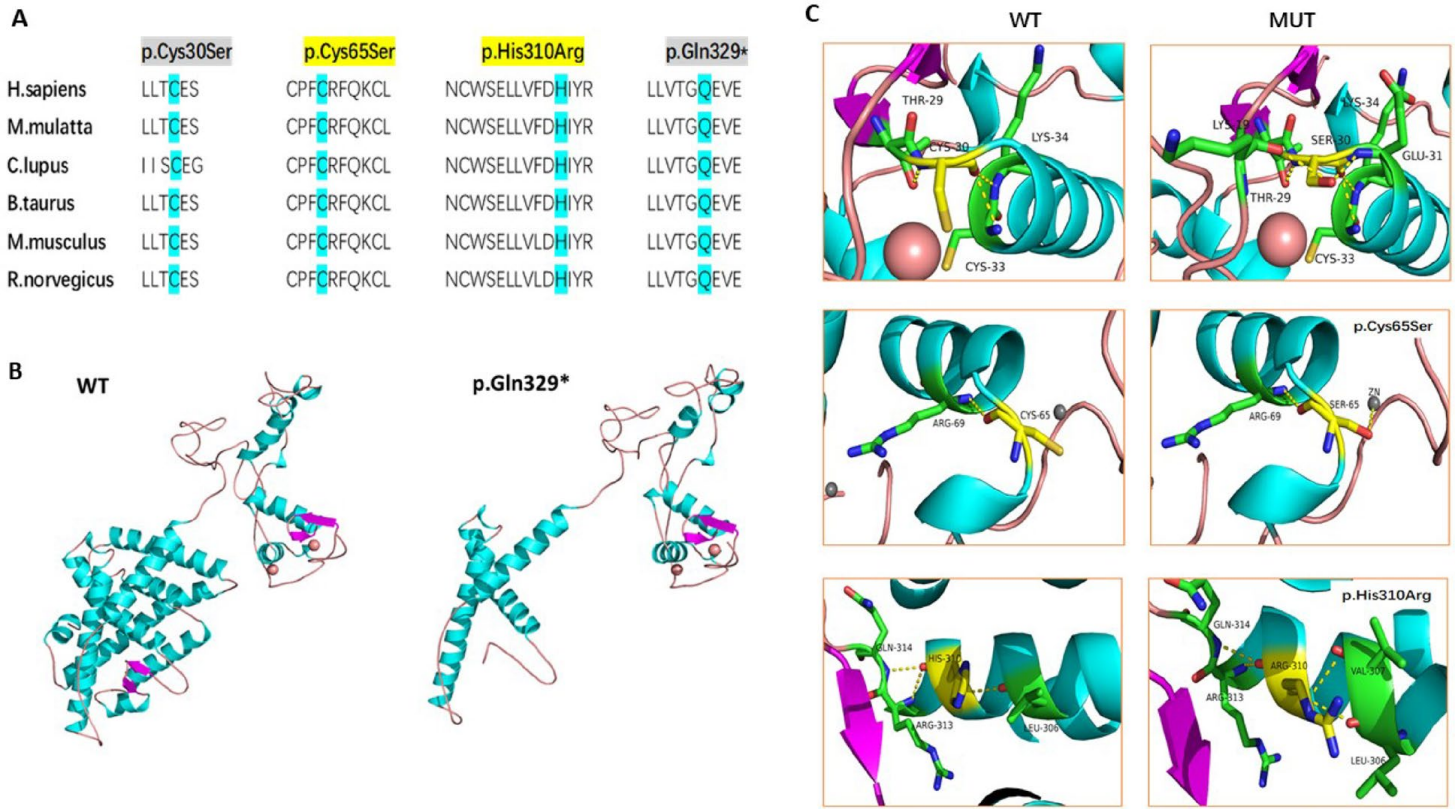
Conservation and structural analysis of the four *NR5A1* variants. (**A**) Multiple sequence alignment of NR5A1 protein sequences across six species, highlighting the positions of the four patient-derived variants. Novel variants are shown in yellow. (**B**) Predicted three-dimensional structures of the wild-type NR5A1 protein and the p.Gln329* truncating variant, modeled using SWISS-MODEL. (**C**) Local structural environments of the wild-type and mutant forms of *NR5A1* for each of the three missense variants, visualized using PyMOL

To further investigate the molecular consequences of these clinically relevant NR5A1 variants, we next assessed their evolutionary conservation and modeled their structural impacts. Conservation analysis of the four NR5A1 variant sites (with novel variants highlighted in yellow) showed that all affected residues are highly conserved across species (Fig. 1A). Structural modeling revealed that the p.Gln329* nonsense variant causes the loss of two β-sheets and six α-helices, significantly disrupting the spatial structure of the NR5A1 protein. It stops translation early resulting in a truncated protein lacking 132 amino acids from the C-terminus, including part of the LBD and the entire AF-2 domain (Fig. 1B).

The p.Cys30Ser variant replaces cysteine with serine, which are both non-charged and non-polar. While the main-chain hydrogen bonding remains unchanged, the substitution of the cysteine side-chain sulfhydryl group with a hydroxyl group allows new hydrogen bonds to form with Lys19 and Glu31. The p.Cys65Ser variant replaces a polar, uncharged cysteine with threonine, which is non-polar and uncharged. Although this change does not disrupt the hydrogen bond with Arg69, it introduces a polar interaction with Zn ions.

Finally, the p.His310Arg variant substitutes histidine—a positively charged, polar residue—with arginine, which is larger but similarly charged. This change preserves the hydrogen bonds with Arg313, Gln314, and Leu306, but introduces a new hydrogen bond with Val307 (Fig. 1C).

### Effects of *NR5A1* variants on protein expression and nuclear localization

**Fig. 2:**
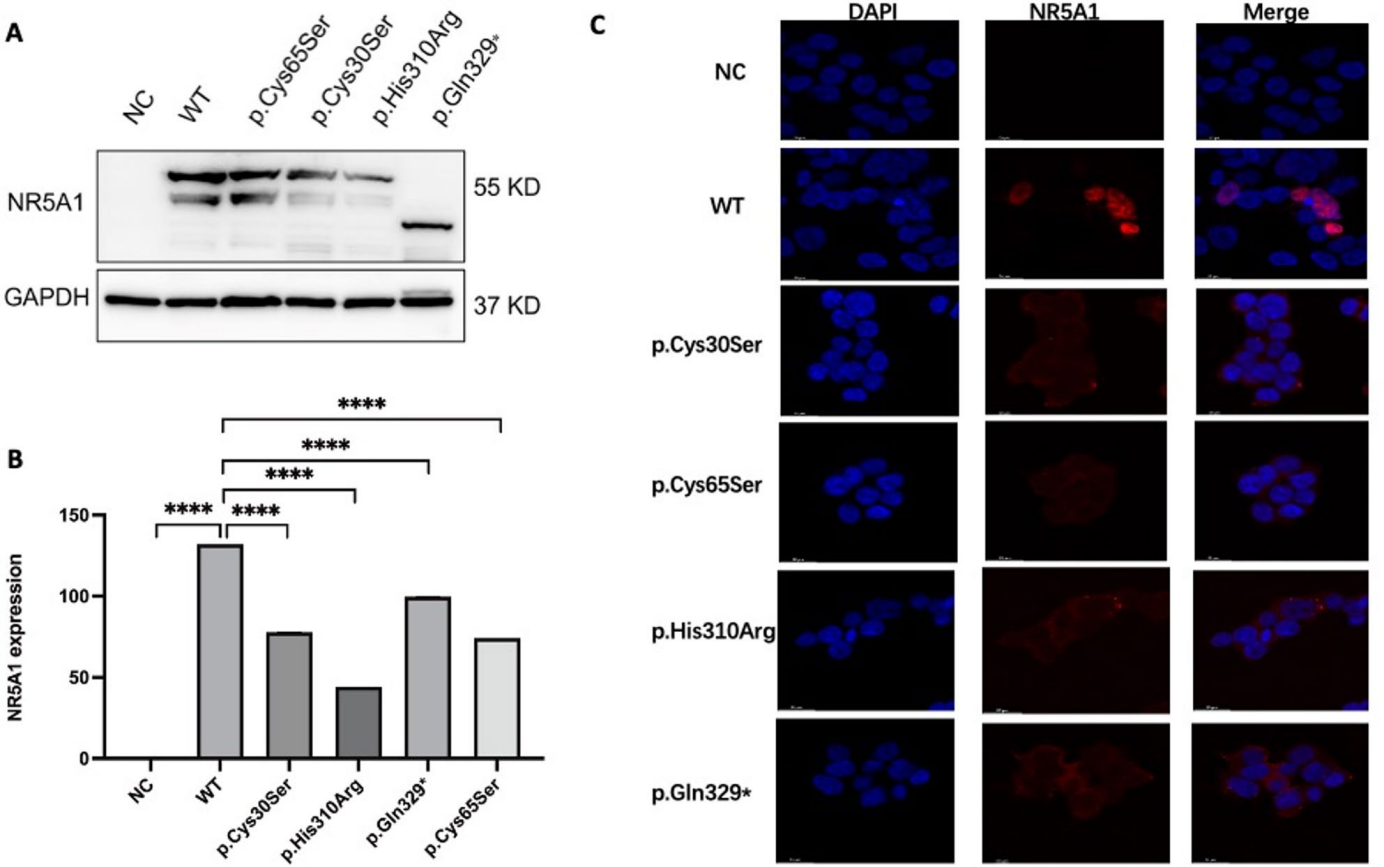
Effects of *NR5A1* variants on protein expression and subcellular localization. (**A**) Western blot analysis showing NR5A1 protein levels in HEK293T cells transfected with wild-type (WT) or mutant *NR5A1* constructs. GAPDH was used as a loading control. (**B**) Quantification of relative NR5A1 protein expression levels from western blot data. Bars represent mean ± SD; ****p < 0.0001. (**C**) Immunofluorescence images of HEK293T cells stained with DAPI (blue) and anti-NR5A1 antibody (red). NC, negative control; WT, wild-type *NR5A1*

To investigate the impact of *NR5A1* variants on protein expression, we transfected HEK293T cells with plasmids encoding either WT or mutant NR5A1 and extracted total protein for western blotting. We successfully detected NR5A1 protein in cells transfected with WT and mutant plasmids, but not with the empty vector, indicating that these cells do not endogenously express NR5A1. Compared with WT NR5A1, all four variants showed reduced protein expression (Fig. 2A and B).

To assess whether these variants affect NR5A1 subcellular localization, immunocytochemistry was performed using laser confocal microscopy. Qualitative assessment of confocal immunofluorescence images from three independent experiments consistently showed that WT NR5A1 localized exclusively to the nucleus, whereas all four variants displayed markedly reduced nuclear signal and increased cytoplasmic distribution (Fig. 2C).

### Transcriptomic effects of the *NR5A1* p.Gln329* variant on steroidogenic pathways

**Fig. 3:**
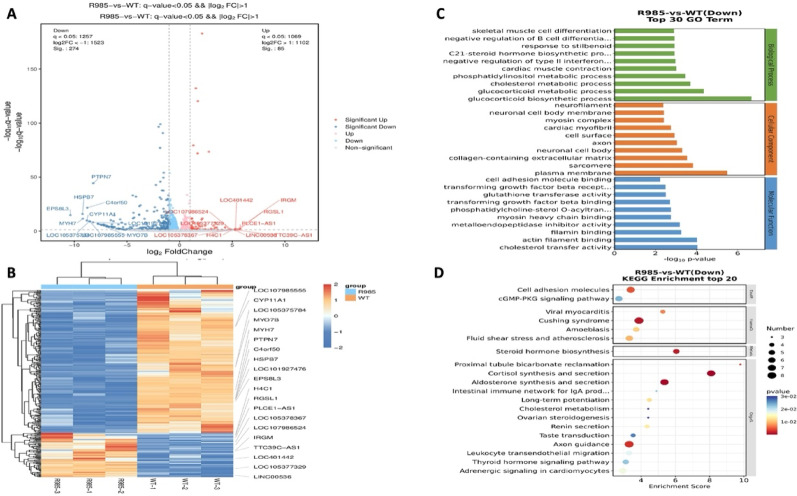
Transcriptomic impact of the *NR5A1* p.Gln329*(R985) variant. (**A**) Volcano plot showing differentially expressed genes in R985-expressing cells versus WT *NR5A1*. Red and blue dots represent significantly upregulated and downregulated genes, respectively (q < 0.05, |log₂FC| > 1). (**B**) Heatmap of the top 30 differentially expressed genes, with clustering based on expression in WT and R985-transfected cells. (**C**) Gene Ontology (GO) enrichment analysis of significantly downregulated genes (biological process, cellular component, molecular function). (**D**) KEGG pathway enrichment analysis of downregulated genes, highlighting steroid hormone biosynthesis and cholesterol metabolism among the top enriched pathways

To explore how NR5A1 gene variants alter downstream gene expression networks, we performed transcriptome sequencing in HEK293T cells transfected with WT or the p.Gln329*. The p.Gln329* variant was selected for the transcriptome (labeled as R985) and CUT&Tag (labeled as C985) analysis for its strongest effect in earlier assays and clinical relevance. Given NR5A1’s central role as a transcriptional regulator of steroidogenic and sex-determining genes, transcriptomic profiling allows for systematic identification of target genes and signaling pathways disrupted by each variant. Transcriptome sequencing identified 359 differentially expressed genes in cells over-expressing *NR5A1* p.Gln329*(R985) variants, including 85 upregulated and 274 downregulated genes (Fig. 3A). Cluster analysis highlighted the top 20 most affected genes, among which *CYP11A1*, a key regulator of sexual and adrenal development, showed significant downregulation (Fig. 3B).

GO enrichment analysis revealed that differentially expressed genes were primarily associated with steroid biosynthesis, steroid metabolism, and cholesterol metabolism in the biological process category. In the cellular component category, affected genes were enriched in membrane-associated components. In terms of molecular function, most were involved in cholesterol transport and transfer (Fig. 3C).

KEGG analysis further confirmed significant enrichment of downregulated genes in pathways related to cortisol synthesis, aldosterone production, and overall steroid metabolism (Fig. 3D), consistent with a functional impact of *NR5A1* variants on steroidogenic gene regulation.

### The *NR5A1* p.Gln329*(C985) variant disrupts DNA binding and candidate target gene regulation

**Fig. 4:**
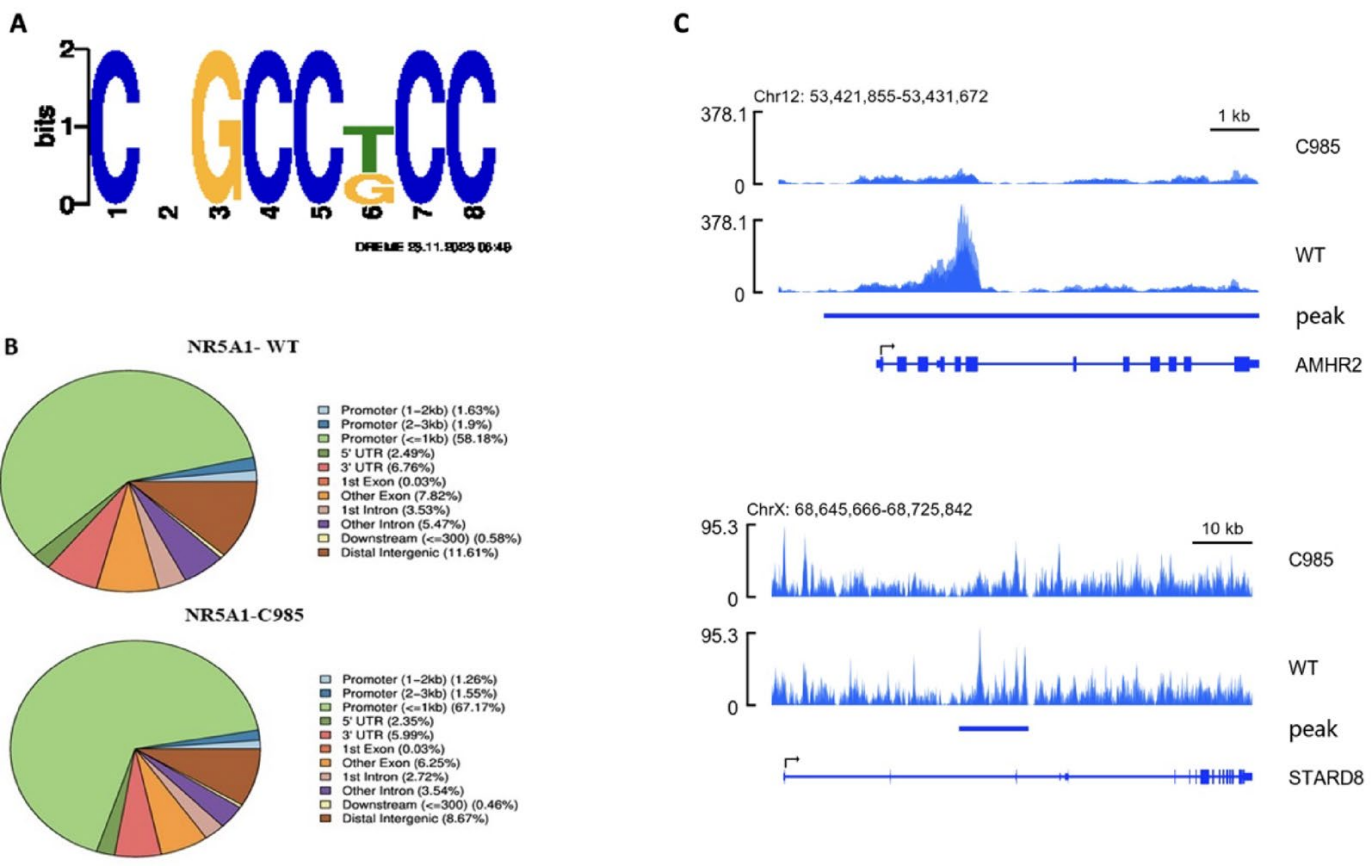
Altered genome-wide DNA binding of the *NR5A1* p.Gln329* (C985) variant. (**A**) *NR5A1* consensus DNA-binding motif identified by CUT&Tag analysis. (**B**) Genomic distribution of *NR5A1* binding peaks in HEK293T cells expressing wild-type (WT) or C985 variant, noting the global proportional increase in promoter-annotated peaks in the mutant, which contrasts with the locus-specific loss of signal at key targets. (**C**) Genome browser tracks showing representative NR5A1 binding at specific loci. WT *NR5A1* shows clear enrichment at target regions, which is markedly reduced in the C985 mutant

NR5A1 regulates gene transcription by binding to specific DNA motifs within target gene promoters. These binding sites often contain conserved short sequences critical for recognition and regulatory activity [12]. To investigate this, we performed motif analysis of CUT&Tag data, revealing enrichment of a “CGCC” sequence, which may represent a consensus NR5A1-binding element (Fig. 4A). Genome-wide mapping showed that NR5A1 binding peaks were predominantly enriched in promoter regions. While the proportion of peaks annotated to promoters was globally higher in the p.Gln329* mutant samples than in WT, this likely reflects a redistribution of residual binding rather than increased specific activity. In contrast, locus‑specific analysis revealed markedly reduced enrichment at the *AMHR2* and *STARD8* loci in the mutant condition (Fig. 4C).

Among the genes downregulated in our RNA-seq analysis were several known NR5A1 targets implicated in sexual development, including *CYP11A1*, *STAR*, *CYP17A1*, and *CYP21A2*. In addition, two strongly downregulated genes (|log₂(fold change)| ≥ 1.5), namely Anti-Müllerian hormone type 2 receptor (*AMHR2*) and STAR-related lipid transfer domain containing 8 (*STARD8*), emerged as strong candidate novel downstream targets of NR5A1 (Table 3). Notably, CUT&Tag sequencing showed reduced genomic enrichment of *AMHR2* and *STARD8* (Fig. 4C). We next aimed to validate downregulation of *AMHR2* and *STARD8*. To do so, we conducted qPCR.

**Table 3:** **Differential expression of** ***AMHR2*** **and** ***STARD8*** **in cells overexpressing the** ***NR5A1*** p.Gln329* (C985)variant

Gene Name	FoldChange	log2FoldChange	*p*-value	q-value	Regulation
*STARD8*	0.195938291	-2.351528735	1.63E-25	7.99E-23	downregulated
*AMHR2*	0.343641092	-1.541025532	1.56E-07	7.90E-06	downregulated

FoldChange and log₂FoldChange represent expression changes in mutant vs. wild-type samples. p-value and q-value indicate statistical significance and false discovery rate, respectively

### qPCR validation confirms downregulation of *AMHR2* and *STARD8*

**Fig. 5:**
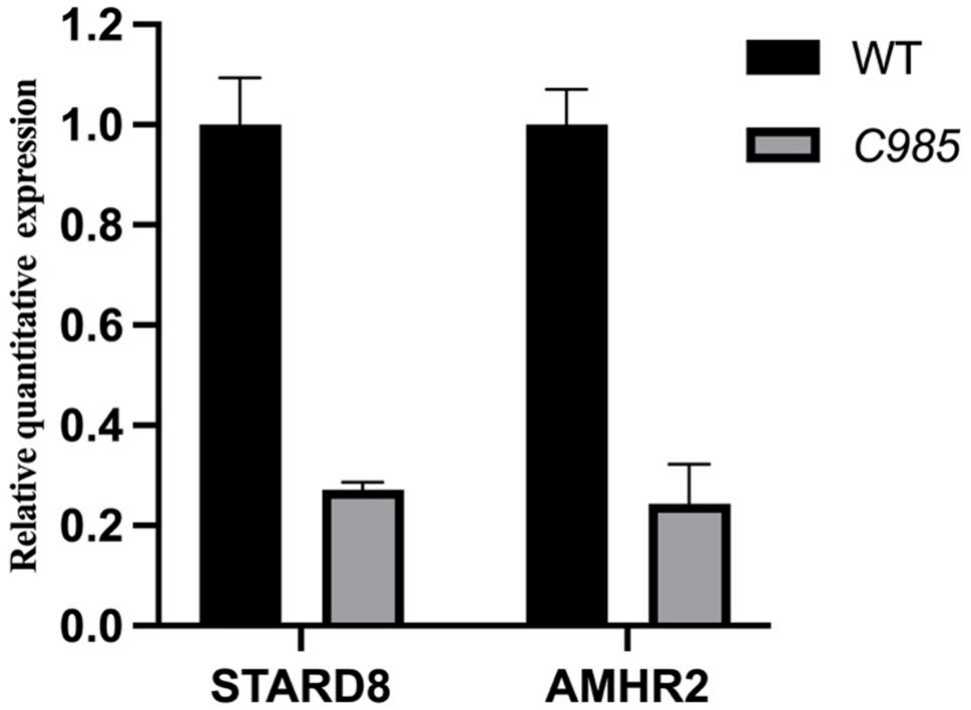
qPCR validation of candidate target gene expression in cells expressing *NR5A1* p.Gln329 variant. Relative mRNA expression levels of *STARD8* and *AMHR2* were significantly reduced in HEK293T cells transfected with the p.Gln329* (C985) mutant compared to wild-type (WT) *NR5A1*, confirming transcriptomic findings. Data are presented as mean ± SD

Consistent with our RNA-seq data, quantitative PCR analysis confirmed that *AMHR2* and *STARD8* mRNA levels were decreased in cells expressing mutant NR5A1 compared with WT (Table 4). When integrated with the CUT&Tag data, we conclude that *AMHR2* and *STARD8* are candidate transcriptional targets of NR5A1 (Fig. 5).

**Table 4:** **Quantitative PCR validation of** ***AMHR2*** **and** ***STARD8*** mRNA expression levels in cells expressing the NR5A1 p.Gln329* (C985)variant

Gene	WT expression level ± standard deviation	C985 expression level ± standard deviation	*P* value
*STARD8*	1 ± 0.093	0.271 ± 0.015	3.36E-05
*AMHR2*	1 ± 0.07	0.243 ± 0.079	0.001

### *NR5A1* variants impair promoter activation of key candidate target genes

**Fig. 6:**
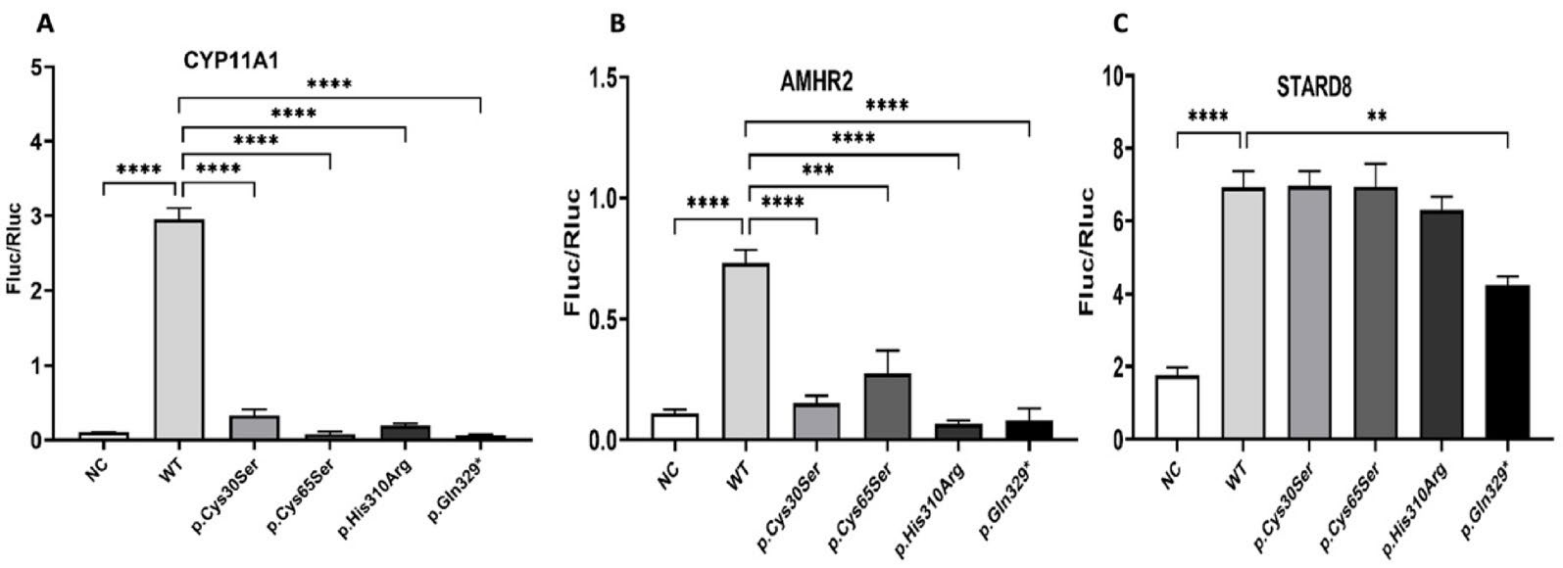
Impact of *NR5A1* variants on the promoter activity of candidate target genes. Dual-luciferase assays showed that all four *NR5A1* variants significantly reduced transcriptional activation of *CYP11A1* (**A**) and *AMHR2* (**B**) promoters compared with wild-type. For *STARD8* (**C**), only the p.Gln329* variant impaired promoter activity. Data are mean ± SD; P values from one-way ANOVA. NC: negative control; WT: wild-type

To functionally validate *NR5A1*-mediated transcriptional regulation of candidate target genes, we performed dual-luciferase reporter assays using the promoter regions of *CYP11A1*, *AMHR2*, and *STARD8*. These three genes were selected based on combined transcriptomic and CUT&Tag analyses, which revealed consistent downregulation of their expression and enrichment of *NR5A1* binding at their promoter regions. Dual luciferase reporter assays demonstrated that WT NR5A1 significantly increased promoter activity compared with the empty vector (*P* < 0.0001). By contrast, all four *NR5A1* variants led to a significant reduction in the transcriptional activity of *CYP11A1* and *AMHR2* promoters (Fig. 6A and B). For the *STARD8* promoter, the p.Cys30Ser, p.Cys65Ser, and p.His310Arg variants did not significantly alter luciferase activity, indicating no effect on its transcriptional regulation; however, the p.Gln329* nonsense variant caused a marked decrease in *STARD8* promoter activity, suggesting a variant-specific loss of regulatory function (Fig. 6C).

## Discussion

The *NR5A1* gene encodes the nuclear receptor steroidogenic factor-1 (SF-1) that regulates adrenal and reproductive development. NR5A1 plays a central role in gonadal development and steroid hormone biosynthesis, and its disruption is increasingly recognized as a key contributor to 46,XY disorders of sex development (DSD). However, the mechanisms by which NR5A1 variants lead to such a broad spectrum of phenotypes remain poorly understood, limiting our ability to predict clinical outcomes or guide personalized treatment strategies. In this study, the consistent presentation of severe proximal hypospadias, bifid scrotum, and inguinal cryptorchidism in all four patients points to a common phenotypic consequence of NR5A1 disruption affecting early testicular development. The absence of isolated genital anomalies and uniformly low EMS values suggest that NR5A1 variants may exert coordinated effects on both Leydig and Sertoli cell function. The presence of undescended testes may result from reduced insulin-like peptide 3, which promotes testicular descent [13], and decreased androgen (testosterone and dihydrotestosterone) levels required for inguinal descent.

AMH, secreted by Sertoli cells, mediates regression of Müllerian structures in male embryos. Its expression is regulated by *SRY* and *SOX9*, with further activation via *NR5A1*, *GATA4*, and *WT1*. While some patients carrying *NR5A1* variants show low AMH levels at birth, others have normal levels, and ~ 17% retain Müllerian structures such as a uterus or vagina [14]. FSH levels tend to rise significantly at puberty and continue to increase over time, even in the absence of Müllerian remnants. Regardless of external genitalia or gender assignment, patients exhibit low serum AMH and inhibin B levels, although most lack detectable Müllerian structures [15]. This finding could point to impaired AMH signaling downstream, despite apparently normal hormone levels — particularly relevant for patients with normal early development but declining inhibin B and elevated FSH over time. This finding suggests that AMH production during embryogenesis remains sufficient and that *NR5A1* variants more strongly affect Leydig cell steroidogenesis than Sertoli cell function. Collectively, these findings indicate that *NR5A1* variants disrupt hormone production, particularly testosterone, insulin-like peptide 3, and AMH, thus contributing to the diverse and variable presentation of 46,XY DSD. NR5A1 variants may exert temporally dynamic or cell-type–specific effects, with stronger impacts on Leydig vs. Sertoli cell function depending on developmental stage. The downregulation of *AMHR2* in variant-expressing cells may help explain some of the discordance between normal AMH levels in infancy and later Sertoli cell dysfunction observed clinically.

The pathogenicity of *NR5A1* variants is closely linked to their location within specific protein domains. NR5A1 belongs to a small class of nuclear receptors that bind DNA as monomers and recognize the PyCAAGGTCA binding motif. The A-box region contributes to DNA binding stability and may facilitate interactions with other transcription factor factors. Adequate levels of functional NR5A1 are crucial for adrenal and gonadal development [16], with proper gene dosage required for transcriptional activation of physiological target genes [17]. The amino terminal region of NR5A1 contains two classic zinc finger DNA-binding domains (Zn1 and Zn2), which are required for high-affinity DNA binding [18].

Here, we showed that the p.Cys30Ser and p.Cys65Ser were located in Zn1 and Zn2, respectively, and likely impair DNA binding. Protein expression analysis also revealed reduced levels and a cytoplasmic localization pattern. As NR5A1 must localize to the nucleus to function as a transcriptional regulator [19], this mislocalization likely underpins gonadal dysfunction. The LBD domain regulates NR5A1 function through interaction with small phospholipids and upstream signaling pathways [20]. It also contains the AF-2 domain, which is critical for co-activator recruitment. The variants p.His310Arg and p.Gln329*, located in the LBD domain, similarly led to reduced protein expression and nuclear exclusion, indicating impaired LBD function. Although DBD variants are often associated with more severe phenotypes [21], some studies suggest that LBD variants can be more deleterious [22]. Our data are in agreement with previous studies suggesting that *NR5A1* variants can differentially affect steroidogenic pathways and gonadal development depending on their domain location. However, our sample size was limited, and building a larger, curated database linking *NR5A1* variants to detailed phenotypic outcomes would support more accurate diagnosis and prognosis in 46,XY DSD, and could ultimately refine variant interpretation under clinical guidelines.

In this study, transcriptomic analysis revealed that *NR5A1* variants caused broad downregulation of genes involved in steroid biosynthesis and metabolism, and cholesterol metabolism. Cholesterol, a precursor for steroid hormones, is synthesized through NR5A1-regulated pathways, with its initial conversion mediated by mitochondrial P450 side-chain cleavage enzyme [23]. The regulatory role of NR5A1 in cellular metabolism, underlies its impact on cell differentiation, proliferation, and hormone production. Loss of NR5A1 leads to a proliferative defect in fetal Leydig cells, reduced steroid production, and absence of androgen-dependent structures, such as seminal vesicles and epididymis. In our study, genes such as *CYP11A1*, *STAR*, *CYP17A1*, and *CYP21A2* were markedly downregulated, all of which are critical for normal Leydig cell function and sexual development. Although early studies described *NR5A1* variants causing both adrenal insufficiency and gonadal dysgenesis [24], later reports showed that most patients with heterozygous variants have isolated gonadal dysfunction without adrenal involvement [25]. This is consistent with animal studies showing that only homozygous *Nr5a1* knockout mice exhibit adrenal insufficiency accompanied by gonadal dysgenesis, while heterozygous mice retain adrenal function but develop gonadal abnormalities [26]. Similarly, the patients in this study showed significant downregulation of steroidogenic genes, but preserved adrenal function, suggesting that human gonadal dysfunction may be more sensitive to NR5A1 haploinsufficiency than adrenal development. Therefore, adrenal insufficiency associated with *NR5A1* variants remains relatively rare.

We also identified significant downregulation of *AMHR2* and *STARD8* expression in cells expressing *NR5A1* variants. Promoter activity assays further demonstrated that *NR5A1* variants significantly impair *AMHR2* transcriptional activation. Mutations in *AMHR2* are linked to persistent Müllerian duct syndrome, a form of abnormal sexual development in 46,XY individuals. Meanwhile, *STARD8* has been implicated in 46,XY gonadal dysgenesis in a case report involving affected sisters [27], suggesting that both genes may be directly regulated by NR5A1. For *STARD8*, only the nonsense variant (p.Gln329*) reduced promoter activity, while the missense variants had no effect, likely due to the greater functional disruption caused by protein truncation. *AMHR2* encodes the anti-Müllerian hormone type 2 receptor, and its dysfunction is known to cause Müllerian duct syndrome [28], which may explain the presence of Müllerian structures in a subset of 46,XY DSD patients. *STARD8*, which contains a steroidogenic acute regulatory-related lipid transfer (StART) domain, has also emerged as a candidate gene in testicular development. A previous case report described two sisters carrying *STARD8* variants associated with 46, XY gonadal dysgenesis [27], although direct functional evidence was lacking. Subsequent studies, along with the present findings, support a role for *STARD8* in male gonadal development [29]. In this study, *STARD8* was significantly downregulated in the context of *NR5A1* mutation, and CUT&tag analysis suggested reduced NR5A1 binding near the *STARD8* locus, supporting a regulatory link. Additionally, NR5A1 may contribute to lineage specification by interacting with other transcription factors during cell reprogramming. Although NR5A1 cannot promote differentiation of pluripotent stem cells into steroidogenic cells, it is not sufficient on its own [30], suggesting that other proteins, such as *STARD8*, may cooperate in this process. Our integrated multi‑omics analysis suggests that *AMHR2* and *STARD8* are strong candidate transcriptional targets of NR5A1. However, we acknowledge that CUT&Tag shows physical association rather than direct functional binding, and the luciferase assays were performed in a non‑physiological cell line. Definitive proof of direct regulation will require future experiments such as ChIP‑qPCR in gonadal cell lines and mutation of the putative NR5A1 binding elements.

In summary, our data indicate that *CYP11A1 is a known target and AMHR2 is a strong candidate target of* NR5A1, while *STARD8* may represent a context‑dependent target, particularly sensitive to the severity of the variant. The phenotypic variability in 46,XY DSD may partially reflect the differential impact on such downstream gene networks.

### Limitations

Several limitations of this study should be acknowledged. First, all functional and transcriptomic experiments were performed in HEK293T cells, which do not express *NR5A1* endogenously and lack the native gonadal chromatin context; therefore, the identified candidate targets require validation in more physiologically relevant cell models. Second, the transcriptomic and CUT&Tag analyses were restricted to the p.Gln329* truncating variant; the transcriptome‑wide consequences of the missense variants remain to be defined. Third, the assessment of subcellular localization was qualitative, and formal quantification of nuclear‑to‑cytoplasmic fluorescence ratios should be pursued in future studies. Finally, the small sample size limits robust genotype‑phenotype correlation, which will require larger multi‑center cohorts.

## Supplementary Information

Supplementary material 1.

Supplementary material 2.

Supplementary material 3.

## Data Availability

The raw sequence data reported in this paper have been deposited in the Genome Sequence Archive (Genomics, Proteomics & Bioinformatics 2025) in National Genomics Data Center (Nucleic Acids Res 2025), China National Center for Bioinformation / Beijing Institute of Genomics, Chinese Academy of Sciences (GSA-Human: HRA014774) that are publicly accessible at https://ngdc.cncb.ac.cn/gsa-human [31] [32]. All data generated or analyzed during this study are included in this published article. Additional datasets used or analyzed during the current study are available from the corresponding author upon reasonable request.

## Abbreviations

AMH: Anti-Müllerian hormone
AMHR2: Anti-Müllerian hormone type 2 receptor
AF: Activation function
Cut&Tag: Cleavage Under Targets and Tagmentation
CYP11A1: Cytochrome P450, family 11, subfamily A, polypeptide 1
DBD: DNA-binding domain
DSD: Disorders of sex development
DHT: Dihydrotestosterone
EMS: External Masculinization Score
FSH: Follicle-stimulating hormone
HCG: Human chorionic gonadotropin
LH: Luteinizing hormone
LBD: Ligand-binding domain
NR5A1: Nuclear receptor subfamily 5 group A member 1
PCR: Polymerase Chain Reaction
KEGG: Kyoto Encyclopedia of Genes and Genomes
SF-1: Steroidogenic factor-1
SRY: Sex determining region Y
STAR: Steroidogenic acute regulatory protein
STARD8: STAR related lipid transfer domain containing 8
WT: Wild type
