# Supplementary material for: Functional and transcriptomic insights into 46,XY disorders of sex development associated with NR5A1 gene variants
Source: Biol Sex Differ. 2026 Jun 9;17:145. doi: 10.1186/s13293-026-00939-0 (PMC13471489; doi:10.1186/s13293-026-00939-0)
Supplement: Supplementary file 1 [file 13293_2026_939_MOESM1_ESM.pdf]

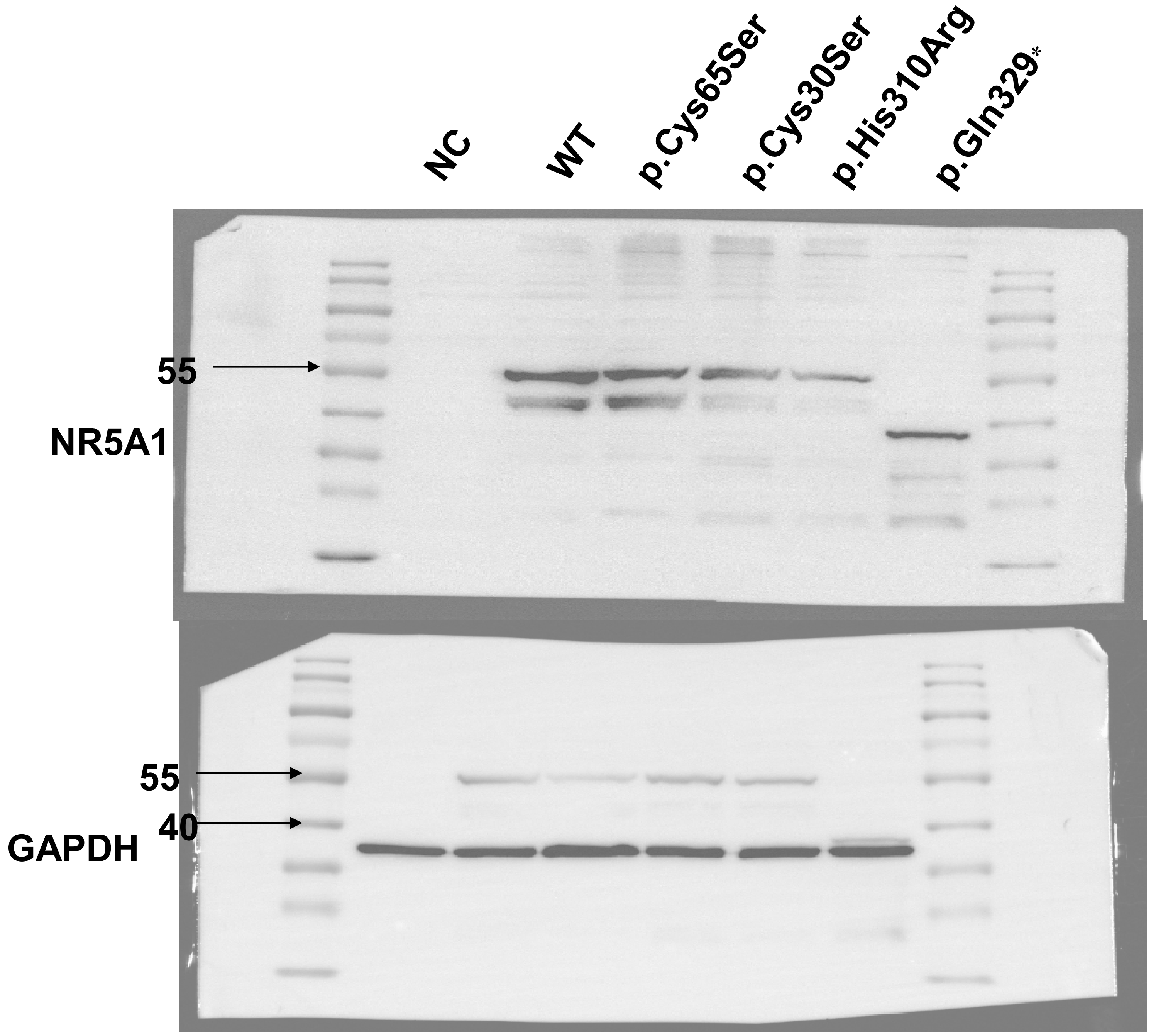
NC
WT
p.Cys65Ser
p.Cys30Ser
p.His310Arg
p.Gln329*
55
NR5A1
55
40
GAPDH
