# Supplementary material for: Functional and transcriptomic insights into 46,XY disorders of sex development associated with NR5A1 gene variants
Source: Biol Sex Differ. 2026 Jun 9;17:145. doi: 10.1186/s13293-026-00939-0 (PMC13471489; doi:10.1186/s13293-026-00939-0)
Supplement: Supplementary file 2 [file 13293_2026_939_MOESM2_ESM.pdf]

伦理审查批件
Approval Letter

上海市儿童医院伦理委员会审查批件　　批件号：2021R092-E01

Approval Letter of Ethics Review Committee, Children's Hospital of Shanghai/
Shanghai Children's Hospital, Shanghai Jiao Tong University　　Approval No: 2021R092-E01

审查日期 Meeting Date	2021 年 8 月 30 日
审查会议地点 Meeting Location	上海市儿童医院<br>Children's Hospital of Shanghai /Shanghai Children's Hospital, Shanghai Jiao Tong University
研究项目名称 Protocol Title	基于遗传分型及内分泌激素水平建立儿童性发育异常多学科精准治疗规范及预后评估体系 To establish a multidisciplinary treatment standard and prognosis assessment system for children with sexual dysplasia based on genetic typing and endocrine hormone levels
项目编号 Protocol No.	2021R092
审查文件 Documents reviewed	■ 伦理审查申请表<br>■ 数据获取表<br>■ 研究方案（1.0 版）<br>■ 知情同意书（法定监护人版）（版本号：1.0/版本日期：2020.8.12）<br>知情同意书（未成年人书面告知版）（版本号：1.0/版本日期：2020.8.12）<br>知情同意书（未成年人口头告知版）（版本号：1.0/版本日期：2020.8.12）<br>■ 主要研究者简历<br>■ 量表
申办者 Sponsor	上海市科学技术委员会
临床研究机构 Clinical Study Unit	上海市儿童医院内分泌科
研究负责人 Principal Investigator	李嫔
伦理审查方式 Review Manner	简易审查/快速审查 Expedited Review（初审）

审查委员 Review Members with Expertise

姓名 Name	职业背景 Profession&Experience	委员角色 Role（注明非机构委员）
徐伟珏	儿科学	委员
孙华君	药学	委员

审查决定 Review Decision

■ 批准 Approved
☐ 修改后批准 Approved with Recommendation
☐ 修改后再审 Resubmission
☐ 暂停或终止已批准的研究 Termination / Suspension
☐ 不批准 Disapproved

审查决定<br>Review Decision	其他：<br>☐ 按原方案进行研究 Research under the original protocol<br>☐ 暂停受试者入组 Suspension of subjects<br><br>注意（请仔细阅读）：<br>1. 临床研究应遵循我国《涉及人的生物医学研究伦理审查办法》、《药物临床试验质量管理规范》、《药物临床试验伦理审查工作指导原则》、《医疗器械临床试验质量管理规范》以及 WMA《赫尔辛基宣言》、CIOMS《人体生物医学研究国际道德指南》、ICH-GCP 等相关法律法规的最新版本的规定。<br>2. 涉及人类遗传资源采集、收集、买卖、出口、出境的研究项目，须在获得人类遗传资源办公室的批件后开展。<br>3. 研究过程中若变更主要研究者，对临床研究方案、知情同意书、招募材料等任何修改，请提交修正案审查申请。<br>4. 研究过程中发生严重不良事件，请及时递交严重不良事件报告，当出现任何可能显著影响试验进行、或增加受试者危险的情况时，请及时向伦理委员会提出书面报告。<br>5. 纳入不符合纳入标准的受试者；研究过程中，符合提前终止的研究标准而没有让受试者退出；给予受试者错误的治疗或不正确的剂量；给予受试者方案禁用的合并用药等没有遵从方案开展研究的情况，请及时递交违背方案报告。<br>6. 暂停或提前终止临床试验，需及时提交暂停/终止研究报告。<br>7. 根据伦理委员会对跟踪审查频率的意见，请在批件失效前 1 个月递交持续审查申请。<br>8. 研究结束时，须向伦理委员会提交结题报告。
伦理审查意见通知<br>Notification	☐附 Attachment ■无 N/A
伦理审查批件有效期<br>Validity of The Approva	2021 年 8 月 30 日- 2022 年 8 月 29 日
伦理委员会联系电话 Tel	021-52976581
伦理委员会主任签字<br>Chairman（signature）	
伦理委员会盖章<br>Ethics Review Committee（seal）	上海市儿童医院 伦理委员会
日期<br>Date	2021 年 8 月 30 日

数据获取内容审查意见

伦理委员会同意本项目在研究过程中能够获取的数据内容如下：

1、研究受试者数或数据获取病例：196 例

2、数据入选标准：诊断为重度尿道下裂患儿

3、数据来源时间段：2021.10-2024.9

4、数据内容获取明细：

1）基本就诊信息	■ 门诊 ■ 住院
2）医嘱信息	■ 门诊处方 ■ 住院医嘱
3）诊断信息	■
4）检验信息	■
5）检查信息	■
6）手术信息	■
7）病历信息	■
8) 体征信息	■
9）其他	□ 其他：__________

受试者隐私信息的管理：医疗信息、病历资料请编码管理，且不能包含任何可关联到个人的任何可识别信息（姓名、出生日期、学校、家庭地址、电话、病例资料号、社保卡号、生物识别信息等）。研究数据请在研究结束后以书面文字及电子档案形式保存 5 年，该数据与受试者和监护人隐私信息的关联编码由上海市儿童医院项目负责人承担双人双锁保管的责任。

本数据仅供研究者院内使用、保存及保管；任何数据提供给申办方、其他第三方，需额外经科研和临床试验管理负责人独立批准，不包含在表中。

请严格按照伦理委员会审查意见进行相关数据的获取和使用，注意个人隐私数据的管理。

本审查意见仅限于内部获取数据审核使用，不作为医院外的使用。

上海市儿童医院伦理委员会

主任委员签字：

日期：2021.8.30
