# Supplementary material for: Functional and transcriptomic insights into 46,XY disorders of sex development associated with NR5A1 gene variants
Source: Biol Sex Differ. 2026 Jun 9;17:145. doi: 10.1186/s13293-026-00939-0 (PMC13471489; doi:10.1186/s13293-026-00939-0)
Supplement: Supplementary file 3 [file 13293_2026_939_MOESM3_ESM.docx]

**Table S1 Primer sequences of *AMHR2*, *CYP11A1* and *STARD8* gene promoters**

| **Gene** | **Upstream primer** | **Downstream primer** | **Length** |
| --- | --- | --- | --- |
| *AMHR2* | TGCAGGTGCCAGAACATTTCTCTATCGATAGGTACCAAGTATAGTATTAAGTGGTAATTTTTTTTCATTGTTGAATTTTG | GCTTACTTAGATCGCAGATCTCGAGCCCGGGCTAGCAGGGCATCCTGGGGCTTCTGATCCCTCAGTCCCCAACTCCG | 2071 bp |
| *CYP11A1* | TGCAGGTGCCAGAACATTTCTCTATCGATAGGTACCTGACCATGGCCATTGGAGAGGATGAGGCAATAACCTCCAGCCCGGGCCACTTCTGGGGAGG | GCTTACTTAGATCGCAGATCTCGAGCCCGGGCTAGCAGCCCTGGGCCACCAGGGCCAAGATTATAACTACCAGCTCAAGGCCATACCAGAAGCTGAT | 2072 bp |
| *STARD8* | TGCAGGTGCCAGAACATTTCTCTATCGATAGGTACCTTAAACCTCCCAAAGGAAGTAGGTACTATTATTATTCCCCATTTTACGGATG | GCTTACTTAGATCGCAGATCTCGAGCCCGGGCTAGCGAGGGAGGGAAAAAAGGAGGGAGGGAGAGAGGCCGGCTAAGGAGGAGGGACCA | 2072 bp |
